# Fluorescent Sensing of Ciprofloxacin and Chloramphenicol in Milk Samples via Inner Filter Effect and Photoinduced Electron Transfer Based on Nanosized Rod-Shaped Eu-MOF

**DOI:** 10.3390/foods11193138

**Published:** 2022-10-09

**Authors:** Xiaoyue Yue, Chaoyun Wu, Zijun Zhou, Long Fu, Yanhong Bai

**Affiliations:** 1 College of Food and Bioengineering, Zhengzhou University of Light Industry, Zhengzhou 450001, China; 2 Henan Institute of Product Quality Supervision and Inspection, Zhengzhou 450047, China; 3 Henan Key Laboratory of Cold Chain Food Quality and Safety Control, Zhengzhou 450001, China

**Keywords:** fluorescent sensing, antibiotic detection, inner filter effect, photoinduced electron transfer, metal organic framework

## Abstract

Rapid, facile, and accurate detection of antibiotic residues is vital for practical applications. Herein, we designed a sensitive, visual, and rapid analytical method for sensitive detection of ciprofloxacin and chloramphenicol based on a nanosized rod-shaped Europium metal organic framework (Eu-MOF). The fluorescent Eu-MOF was firstly synthesized by a simple synthetic route at room temperature, which displays a red emission. The mechanisms of detecting ciprofloxacin and chloramphenicol were confirmed to be the inner filter effect (IFE) and photoinduced electron transfer (PET). Under the optimized experimental conditions, the detection limits of the developed method for ciprofloxacin and chloramphenicol detection were 0.0136 and 3.16 μM, respectively. Moreover, the sensor was effectively applied for quantitative determination of ciprofloxacin and chloramphenicol milk samples with satisfactory recoveries of 94.5–102% and 97–110%, respectively. This work developed a new method for rapid detection of ciprofloxacin and chloramphenicol residues. In addition, the established method has potential practical application value for on-site safety regulation on antibiotic residues in animal-derived food.

## 1. Introduction

As people pay increasingly more attention to environmental protection and public food safety, detection of ultra-trace pollutants in nature carries great significance to the construction of ecological civilization and sustainable development. Thereinto, the problem of antibiotic residues recently became a social problem in most countries, which should be resolved. Antibiotics are powerful weapons against infectious bacteria, which have been widely used in animal husbandry to prevent pathema and promote growth. Undoubtedly, rational use of existing antibiotics has effectively increased the production and revenue of animal husbandry and aquaculture industries. Nevertheless, their unreasonable abuse and excessive residues have also lead to catastrophic pollution in animal-derived food as well as water and soil resources, which threatens human health [1]. Therefore, it is particularly important for us to establish rapid, sensitive, and selective methods to detect antibiotic residues [2]. Ciprofloxacin is the most used fluoroquinolone drug for the treatment of pneumonia, lower respiratory tract infections, and skin infections. However, ciprofloxacin has slow metabolism and degradation, whose abuse can lead to excessive ciprofloxacin residue in animal-derived foods [3]. Besides, ciprofloxacin residues can cause a series of problems, including allergic reactions, intestinal flora disorders, antibiotic resistance, posing a threat to human health [4]. Chloramphenicol is often widely used as a cheap and effective veterinary drug in the field of animal husbandry. Chloramphenicol can bind to the 70S ribosome of human mitochondria, thus inhibiting protein synthesis in human mitochondria and causing aplastic anemia and visual impairment in humans [5,6]. At present, the United States, the European Union, China, etc., stipulate that chloramphenicol should not be detected in animal-derived foods [5,7]. Therefore, it is our inevitable choice to establish an efficient and sensitive method for the detection of ciprofloxacin and chloramphenicol.

There are many well-established antibiotic testing technologies to choose from, such as capillary electrophoresis [8], high-performance liquid chromatography [9], ion mobility spectrometry, Raman spectroscopy [10], and liquid chromatography-tandem mass spectrometry [11,12]. However, most of these methods have the disadvantages of dependence on expensive instruments, complicated operations, and need for necessary sample pretreatment. Therefore, it is necessary to establish a new and simple antibiotic detection method [13].

Fluorescent sensors enjoy the superiorities of high sensitivity, easy accessibility, and low cost, which means great attraction in detection. Generally, fluorescent sensors need to use excellent fluorescent probes as the signal platform to achieve the sensitive detection of targets. Nowadays, there have been many attempts to construct fluorescent sensors using different kinds of fluorescent materials as much as possible, which mainly include fluorescent carbon dots (CDs), II–VI semiconductor quantum dots, and metal organic frameworks (MOFs) materials. Thereinto, as a promising new type of crystalline material, metal organic frameworks (MOFs) materials are widely used in research fields, such as biochemical catalysis, gas storage, bioimaging, drug delivery, and chemical sensing. With unique advantages in chemical sensing, such as narrow fluorescence emission, high porosity, structural and chemical tunability, large Stokes shift values, rapid response, high sensitivity and selectivity, luminescent europium metal-organic framework (Eu-MOF) has become a smart material, that has attracted intense attention in the past few decades [14,15]. However, most of these experiments are often based on a single transmission signal, which is very susceptible to environmental interference, resulting in unstable experimental signals, which is not conducive to multiple experiments. Compared to the single fluorescence-based method, dual emission fluorescence sensors can minimize these effects and achieve higher analysis accuracy by self-calibrating the two fluorescence intensities. Generally speaking, with perceptible color change, dual emission fluorescence sensors support quick visual recognition. Therefore, the use of MOF-based dual emission ratio fluorescence sensors enjoys more promising prospects [16]. Due to unknown factors, such as environment, instrumentation, etc., single-channel fluorescence sensors typically have lower reliability and selectivity [17]. Dual emission fluorescence sensors are receiving increasing attention due to their advantages of eliminating background noise and improving signal-to-noise ratios [18,19]. Dual emission fluorescence probes rely on changes in the intensity of two or more emission bands caused by the analyte, by this method, the results may be more accurate and sensitive [20].

The sensing mechanisms observed in luminescent metal-organic frameworks materials generally include ligand metal energy transfer (LMET), fluorescence (Förster) resonance energy transfer (FRET), photoinduced electron transfer (PET), antenna effect, inner filter effect (IFE), skeleton collapse and adsorption [21,22,23]. The photoelectron property of europium ions is a result of 4f electrons, including long fluorescence lifetime, and sharp and stable emission bands. In addition, due to the “antenna effect”, electrons of the ligand are transferred to the europium ions, which increases the luminous intensity. These characteristics greatly expand the application scope and development potential of Ln-MOF. At present, the application of Ln-MOFs in temperature sensing, small molecule sensing, metal ion sensing, and anion sensing has been widely reported [24].

In this work, a rapid fluorescence detection method was established for the detection of ciprofloxacin and chloramphenicol. First, Eu-MOF with a red fluorescent emission was synthesized. Eu-MOF consists of Eu^3+^ as a metal node and 1,3,5-benzenetricarboxylic acid, which acts like a bridge, causing a strong red fluorescence under the stimulation of excitation light. Based on the inner filter effect and photoelectron-induced transfer, chloramphenicol can quench the fluorescence of Eu-MOF. Moreover, the fluorescence intensity of ciprofloxacin can also be increased due to photoelectron-induced transfer, and the emission peak will also be higher. Therefore, Eu-MOF can be used as single emission and dual emission fluorescence sensors to detect chloramphenicol and ciprofloxacin in food, respectively.

## 2. Experimental

### 2.1. Main Reagents and Materials

Eu (NO_3_)_3_·6H_2_O and trimesic acid (H_3_BTC) were purchased from Aladdin (Shanghai, China). Ciprofloxacin (CIP), chloramphenicol (CHL), erythromycin (ERY), florfenicol (FLO), gentamycin sulfate (GEN), kanamycin Sulfate (KAN), lincomycin hydrochloride (LIN), neomycin (NEO), streptomycin (STR), and thiamphenicol (THI) were from Sangon Biotech Co., Ltd. (Shanghai, China).

### 2.2. Experimental Instruments

The morphology was investigated by scanning electron microscopy using a JSM-7001F. Powder X-ray diffraction (XRD) spectra were recorded using an X-ray device (D8 ADVANCE, Bruker Corp, Karlsruhe, Germany). Fourier transform infrared (FT-IR) spectra were recorded on an Antaris II FTIR spectrophotometer (San Jose, CA, USA). The fluorescence spectrum and the UV-vis absorption spectrum were measured on a Hitachi F-7000 fluorescence spectrophotometer and a TU-1810 spectrophotometer (Beijing Purkinje General Instrument Co., Ltd., Beijing, China), respectively. Fluorescence spectra were obtained from a Hitachi F-7000 fluorescence spectrophotometer (Hiyachi, Tokyo, Japan). The steady-state/transient fluorescence spectra were measured a FLS-980 transient fluorescence spectrophotometer (Livingston, UK). HPLC measurements were performed on an Agilent 1290 Infinity II HPLC (Agilent, Palo Alto, CA, USA).

### 2.3. The Preparation of Eu-MOF

The preparation of Eu-MOF referred to the previous method reported by Li’s group with some modificatione [25]. Specifically, 10 mM Eu (NO_3_)_3_·6H_2_O was dissolved in deionized water and 10 mM trimesic acid was dissolved in absolute ethanol via ultrasonic vibration. Then, the metal precursor was slowly added to the organic ligand solution, and magnetically stirred at room temperature for one hour. After centrifugation at 10,000 rpm min^−1^ for 3 min, the precipitate was washed with water and absolute ethanol, and then, the final product was dried at 60 °C for 6 h.

### 2.4. Fluorescence Detection of Ciprofloxacin and Chloramphenicol

We dispersed 0.004 g of Eu-MOF powder in 10 mL of deionized water by ultrasonic. Then, different concentrations of ciprofloxacin (0.1–18 μM) and chloramphenicol (5–150 μM) were added to the above Eu-MOF solution, respectively. The volume ratio of ciprofloxacin to Eu-MOF solution was 4:1, and the volume ratio of chloramphenicol to Eu-MOF solution was 12:1. The corresponding solution was added to a 3.5 mL quartz fluorescent cuvette, and all fluorescence measurements were repeated three times at the excitation wavelength of 258 nm.

### 2.5. Determination of Ciprofloxacin and Chloramphenicol in Milk

The liquid milk samples were bought from a local supermarket and pretreated according to a national standard of China (GB/T 29692-2013) with some modification. We added 100 μL phosphoric acid and 4 mL acetonitrile to 2 g liquid milk. Then, it was stirred at medium speed for 5 min. Afterwards, the above solution was centrifuged at 10,000 rpm min^−1^ for 10 min, and then transferred the upper supernatant to a beaker for future usage. Five mL n-hexane was added to the supernatant, vortexed for 1 min and left to stand for 5 min. Transferred the residues to a pear-shaped flask. Next, 4 mL of acetonitrile was added into the residue and the extraction was repeated once. The supernatant was combined with the same portion of n-hexane, and the two extracts were combined and rotary evaporated at 50 °C. The residue was dissolved in 50 mL of deionized water, and filtered through a 0.22 μM organic filter membrane for HPLC determination and FL analysis.

## 3. Results and Discussions

### 3.1. Physical Characterization of Eu-MOF

**Figure 1:**
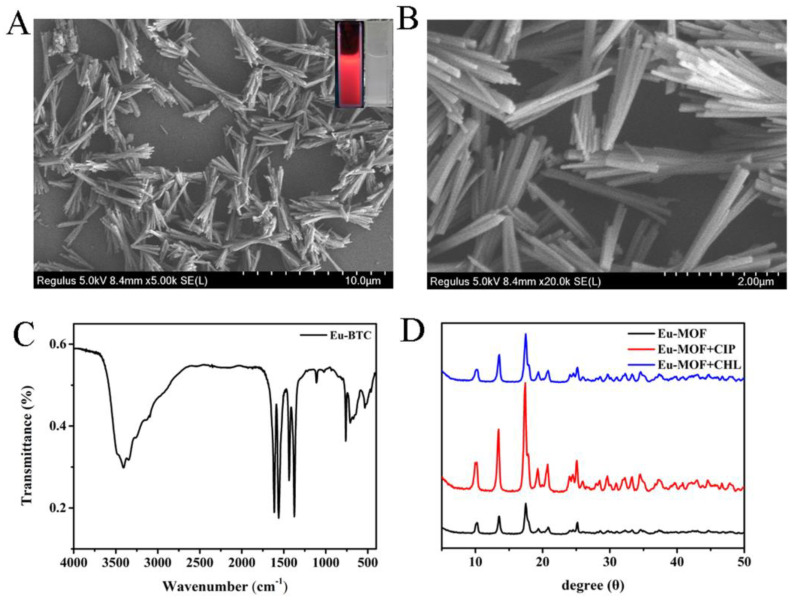
SEM images of Eu-MOF at low magnification (**A**) and high magnification (**B**); (**C**) FTIR spectra of Eu-MOF; (**D**) XRD spectra of Eu-MOF before and after the addition of CIP and CHL; Inset of (**A**): optical images of Eu-MOF solution under a UV lamp (left) and natural light (right).

To deeply understand the properties of the prepared Eu-MOF, SEM, FTIR, and XRD were used to characterize the morphology and structures of Eu-MOF. Figure 1A,B shows that Eu-MOF is a typical nanosized rod-shaped structure with good monodispersity. The illustration is a photo of Eu-MOF solution under ultraviolet light and f natural light, respectively. Eu-MOF is a white translucent suspension under natural light. When the aqueous dispersion of Eu-MOF is exposed to 254 nm ultraviolet light, bright red fluorescence can be easily observed. In addition, the composition of Eu-MOF was further confirmed by FT-IR spectroscopy. As shown in Figure 1C, the strong absorption at 3408 cm^−1^ is attributed to the -OH stretching vibration group of H_2_O, indicating that water molecules act as solvents and reactants at the same time. The characteristic bands appearing in 1613–1560 cm^−1^ and 1436–1374 cm^−1^, respectively, represent the symmetric and antisymmetric stretching vibration of -COO group of the ionizing ligand [26]. The absorption peak at 534 cm^−1^ is attributed to Eu-O stretching vibration, indicating that Eu ion interacts with -COO group [27].

### 3.2. Detection Mechanisms

**Figure 2:**
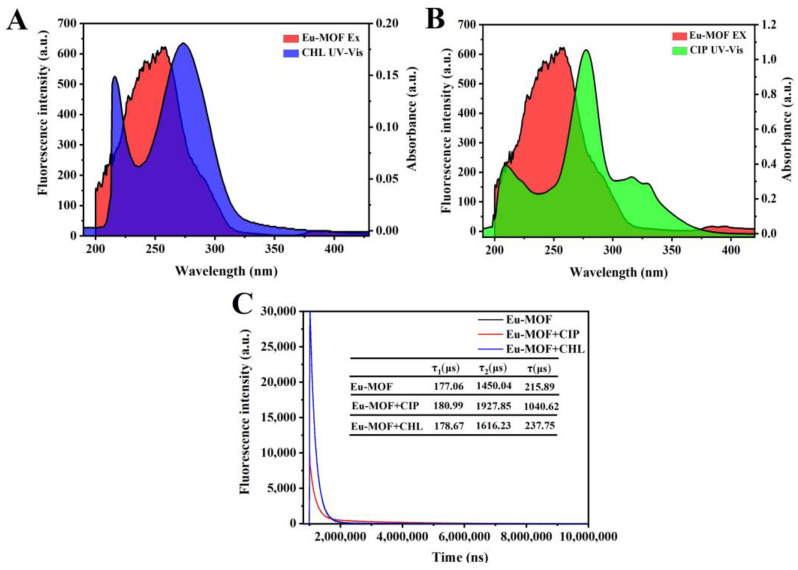
Fluorescence excitation spectra of Eu-MOF overlaid with the UV-vis absorption spectra of ciprofloxacin (**A**) and chloramphenicol (**B**); (**C**) fluorescence lifetime spectra of Eu-MOF before and after the addition of ciprofloxacin and chloramphenicol.

Fluorescence-sensing mechanisms usually include structure collapse, energy absorption, and electron transfer [28,29]. To prove the mechanism for fluorescent detection of ciprofloxacin and chloramphenicol, we first measured the XRD spectra of Eu-MOF. As shown in Figure 1D, the crystal structure of Eu-MOF remains unchanged after the addition of ciprofloxacin or chloramphenicol, indicating that the Eu-MOF skeleton did not collapse, and it was ruled out that the skeleton collapse caused the fluorescence quenching of Eu-MOF. As can be seen from Figure 2A and Figure 2B, the ultraviolet absorption peaks of ciprofloxacin and chloramphenicol almost completely overlap with the fluorescence excitation peaks of Eu-MOF, so the inner filter effect was one of the quenching mechanisms [1]. Later, to confirm the other detection mechanisms, the steady state transient fluorescence spectra of Eu-MOF were obtained in the absence or presence of ciprofloxacin and chloramphenicol. As shown in Figure 2C, the fluorescence lifetime of Eu-MOF does not change after the addition of chloramphenicol. However, the fluorescence lifetime of Eu-MOF increases to some extent after the addition of ciprofloxacin, which may be caused by the blue fluorescence of ciprofloxacin itself. In addition, the quenching time of Eu-MOF is less than 20 s, so the quenching mechanism may not be that antibiotics are adsorbed into the voids of Eu-MOF. Therefore, the inner filter effect was one of the mechanisms for ciprofloxacin and chloramphenicol detection based on fluorescent Eu-MOF.

To analyze whether the detection mechanisms include photoelectron-induced transfer besides the inner filter effect, the highest occupied molecular orbital (HOMO) energy was calculated based on the ultraviolet photoelectron spectroscopy (UPS) of Eu-MOF. HOMO energy was determined by the incident light energy (hυ = 21.2 eV), onset binding energy (E_onset_), and cutoff binding energy (E_cutoff_) [30]. The formula is as follows:

E_HOMO_ = hυ − (E_cutoff_ − E_onset_) (1)

**Figure 3:**
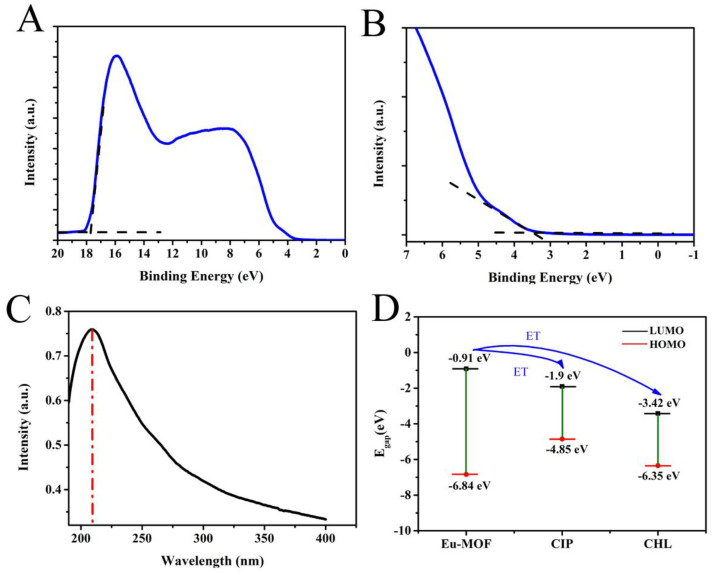
UPS spectra of Eu-MOF in (**A**) the second edge region and (**B**) the HOMO region; (**C**) UV absorption spectra of Eu-MOF; (**D**) HOMO-LUMO energy levels and molecular front orbital energy levels of Eu-MOF and CIP and CHL.

Figure 3 shows the UPS spectrum of Eu-MOF (Figure 3A) and the UV-vis absorption spectra of Eu-MOF (Figure 3C). The abscissa is the binding energy relative to the Fermi energy (EF) of Au, which is defined by the energy of the electron relative to the vacuum level before excitation. Figure 3A shows the high binding energy cutoff (E_cutoff_) of Eu-MOF. E_cutoff_ was calculated according to the linear extrapolation of the secondary electron yield to zero. Figure 3A shows that the high binding energy cutoff of Eu-MOF was 17.75 eV. Figure 3B shows the HOMO area of Eu-MOF. E_cutoff_ = 17.75 eV, E_onset_ = 3.39 eV, so E_HOMO_ can be calculated as −6.84 eV based on the Equation (1). The band gap is 5.93 eV according to the UV absorption spectra of Eu-MOF. The energy of the lowest unoccupied molecular orbital E_HOMO_ = E_LOMO_-band gap, so E_LUMO_ is −0.91 eV [21]. As shown in Figure 3D, E_LUMO_ of ciprofloxacin is −1.9 eV and E_LUMO_ of chloramphenicol = −3.42 eV. Under lower LUMO energy of the analyte, transition of excited state electrons is easier. Simply put, Eu-MOF can be regarded as a large “molecule”. The energy levels of the valence band (VB) and conduction band (CB) can be described in a model similar to molecular orbitals (MOs). The CB energy level of MOF is higher than the lowest unoccupied molecular orbital (LOMO) of the analyte, which results in the driving force for electron transfer from MOF to the analyte, leading to fluorescence quenching [25,31]. Therefore, electrons may be transferred from Eu-MOF to ciprofloxacin and chloramphenicol under light-induced action. From Figure S1, we can clearly see that with the addition of ciprofloxacin to Eu-MOF solution, the fluorescence peak of ciprofloxacin at 449 nm gradually increases and stabilizes at 200 s, which also proves that the electrons of Eu-MOF transferred to ciprofloxacin, leading to the enhanced fluorescence of ciprofloxacin. Therefore, the detection mechanism should be ascribed to a joint result of the inner filter effect and photoelectron-induced transfer.

### 3.3. Optimization of Detection Conditions

The excitation wavelength of Eu-MOF was firstly optimized. As shown in Figure S1A, the fluorescence intensity of Eu-MOF increases with the increasing excitation wavelength. When the excitation light wavelength is 258 nm, the fluorescence intensity reaches the maximum. Afterwards, the fluorescence intensity of Eu-MOF gradually decreases with the increase of excitation wavelength. As the excitation wavelength changes, the emission wavelength does not shift, indicating that the Eu-MOF emission is excitation-independent emission [32,33]. Subsequently, the reaction time was investigated between the Eu-MOF sensor and antibiotics. As shown in Figure S1B,C, the reaction time of ciprofloxacin quenching Eu-MOF is less than 20 s. However, after the ciprofloxacin and Eu-MOF are mixed, its own fluorescence at 449 nm also increases until the fluorescence intensity stabilizes for 200 s. However, similar to ciprofloxacin, chloramphenicol can quench Eu-MOF in less than 20 s, allowing for instantaneous quenching of Eu-MOF fluorescence.

### 3.4. Fluorescence Detection of Ciprofloxacin and Chloramphenicol

Antenna effects are often observed in lanthanide complexes. Organic ligands absorb photons like antennas and generate tristates from single states through crossover between systems. Compared with free ions, the triplet makes lanthanide ions more sensitive and therefore has a brighter emission [16]. The energy gap between the singlet state and triplet state of ligand is key to effective emission, and the energy gap between the triplet state of the ligand and the excited state of Ln^3+^ ion is also strictly limited. Therefore, the luminescence of Eu-MOF is attributed to the energy transfer process from ligand to metal: The ligand absorbs the excited energy, and then transfers to the Eu^3+^ ion, resulting in Eu^3+^ luminescence [24]. Under the excitation of 258 nm, Eu-MOF displays three characteristic emission peaks located at 595, 618.8, and 690 nm, respectively. The maximum emission peak of Eu-MOF appears at 618.8 nm, which is the typical emission peak of Eu^3+^ [25].

**Figure 4:**
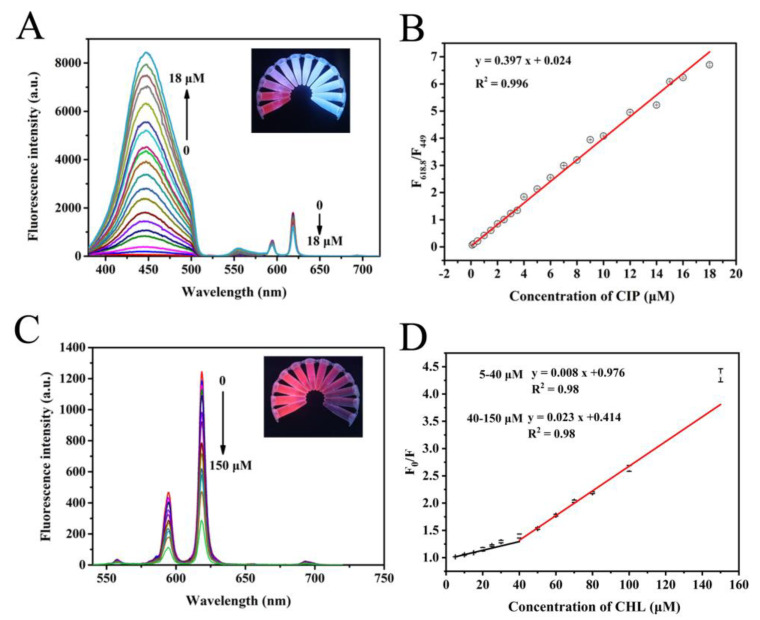
(**A**) Fluorescence spectra and (**B**) standard curve of Eu-MOF under the addition of different concentrations of ciprofloxacin; Inset of (**A**): optical images of fluorescent sensor after addition of different concentrations of ciprofloxacin in centrifuge tubes under a UV lamp at 254 nm; (**C**) fluorescence spectra and (**D**) standard curveof Eu-MOF under the addition of different concentrations of chloramphenicol; Inset of (**C**): optical images of fluorescent sensor after addition of different concentrations of chloramphenicol in centrifuge tubes under a UV lamp at 254 nm.

Under the optimized conditions, the fluorescence sensor based on Eu-MOF was established to detect ciprofloxacin and chloramphenicol. As shown in Figure 4A, as the ciprofloxacin concentration gradually increases, the fluorescence of ciprofloxacin itself at 449 nm gradually increases. While the fluorescence intensity of Eu-MOF gradually decreases, and the fluorescent color of the detection solution shows a clear transition from red to blue (the inset of Figure 4A). Therefore, it is possible to use the ratio of fluorescence intensity at different positions to draw a standard curve. As exhibited in Figure 4B, there was a good relationship between the fluorescence intensity ratio (F_618.8_/F_449_) and the ciprofloxacin concentrations in a wide range of 0.1–18 μM with a lower detection limit of 0.0136 μM. Figure 4C shows the fluorescence spectrum of Eu-MOF after the addition of different concentrations of chloramphenicol. The fluorescence intensity of Eu-MOF gradually decreases with the increasing chloramphenicol concentration, showing obvious brightness transition from bright pink to dark pink. As shown in Figure 4D, there was a good relationship between the fluorescence intensity ratio (F_0_/F) and the chloramphenicol concentrations in a wide range of 5–150 μM with a lower detection limit of 3.16 μM. Two linear ranges displayed in the range of 5–150 μM. The corresponding fluorescent intensity exhibited a linear response toward chloramphenicol concentrations within the range of 5–40 μM, which could be described by F_0_/F = 0.008 c + 0.976. However, the corresponding linear equation could be described by F_0_/F = 0.023 c + 0.414 in the linear range of 40–150 μM. In addition, compared with previously reported CIP and CHL assays (Table 1), the developed fluorescent sensor shows the proposed method displayed comparable or even superior analytical performance.

### 3.5. Anti-Interference Performance

To verify whether other antibiotics can affect the fluorescence of Eu-MOF, streptomycin, kanamycin sulfate, florfenicol, gentamicin, lincomycin hydrochloride, erythromycin, neomycin, and thiamphenicol were used to investigate the anti-interference performance of the developed sensor. As shown in Figure S2A, compared with the blank, the ratio of fluorescence intensity at 618.8 nm to fluorescence intensity at 449 nm is greater than 1 in the presence of only ciprofloxacin (F_618.8_/F_449_). When other antibiotics are added, F_618.8_/F_449_ is basically the same as that of blank, indicating that only ciprofloxacin has a strong emission peak at 449 nm. As shown in Figure S2B, only chloramphenicol can quench the fluorescence of Eu-MOF. Hence, the method for detecting ciprofloxacin and chloramphenicol established in this study has good anti-interference ability.

### 3.6. The Practical Application of Fluorescence Sensor Based on Eu-MOF in the Detection of Ciprofloxacin and Chloramphenicol

In order to explore the accuracy of the constructed fluorescence sensor in practical applications, pure milk purchased from the supermarket was used as the actual sample to detect the concentration of ciprofloxacin and chloramphenicol. As shown in Table 2, the results obtained by this method were consistent with those of high-performance liquid chromatography (HPLC). The results display that the recovery of the developed method for ciprofloxacin detection in milk was 94.5–102%, with a relative standard deviation of 3.6–9.1%. The recovery of the developed method for chloramphenicol detection in milk was 97–110%, with a relative standard deviation of 1.5–4.8%. Therefore, this method could be used for the detection of ciprofloxacin and chloramphenicol in milk.

## 4. Conclusions

In conclusion, a fluorescent sensor based on Eu-MOF was established for the rapid detection of ciprofloxacin and chloramphenicol. Firstly, Eu-MOF was successfully synthesized at room temperature. Then, sensitive, and selective detection of ciprofloxacin and chloramphenicol was achieved via the mechanism integrating the inner filter effect (IFE) and photoinduced electron transfer. The concentration ranges for ciprofloxacin and chloramphenicol detection were 0.1–18 μM and 5–150 μM, with low detection limits of 0.0136 μM and 3.16 μM, respectively. Furthermore, the established method was applied for ciprofloxacin and chloramphenicol detection in actual samples, showing satisfactory reproducibility, stability, and accuracy. Conclusively, the developed method is highly sensitive, selective, and capable of visual detection, which provides ideas for the future on-site visual semi-quantitative detection of antibiotics in food.

## Figures and Tables

**Table 1:** The comparison of Eu-MOF with other methods for CIP and CHL detection.

Methods	Materials	Analytes	Linear Range	LOD	Ref.
Electrochemical	Graphene	Ciprofloxacin	0.1–100 μM	0.1 μM	[34]
Fluorescence	CdSe quantum dots	Ciprofloxacin	0–120 μM	0.6 μM	[35]
Electrochemical		Ciprofloxacin	10–80 µM	0.050 μM	[36]
Fluorescence	Eu^3+^ Doped in Sol-Gel Matrix	Ciprofloxacin	5.0 × 10^−3^–1.0 μM	1.65 × 10 ^−3^ μM	[37]
Colorimetric	Triangular gold nanoplates	Chloramphenicol	0–2000 μM	5 μM	[38]
Electrochemical	Silver chloride/molybdenum disulfide	Chloramphenicol	4–531 μM	1.93 μM	[39]
Fluorescence	Eu-MOF	Ciprofloxacin	0.1–18 μM	0.0136 μM	This work
		Chloramphenicol	5–150 μM	3.16 μM	

**Table 2:** Detection of CIP and CHL in Spiked Milk Samples.

	Spiked Concentration (μM)	Eu-MOF	HPLC	Recoveries (%)	RSD (%)
CIP	2	2.001	2.152	100	3.6
	5	4.728	6.402	94.5	4.2
	10	10.196	12.362	102	9.1
CHL	10	9.704	12.106	97	4.8
	15	16.549	16.508	110	1.5
	20	20.898	20.133	104	3.6

## Data Availability

Data arecontained within the article.

## Supplementary Materials

The following supporting information can be downloaded at: https://www.mdpi.com/article/10.3390/foods11193138/s1, Figure S1: (A) Fluorescence emission spectra of Eu-MOF at different excitation wavelengths; (B) Fluorescence intensity of Eu-MOF at 449 nm and 618.8 nm after the addition of ciprofloxacin with time; (C) Fluorescence intensity of Eu-MOF at 618.8 nm after the addition of chloramphenicol with time; Figure S2: (A) The F449/F618.8 and (B) (F_0_ − F)/F_0_ at 618.8 nm of the Eu-MOF against various antibiotics.

## References

[1] Zhao N., Liu J., Yang F., Lv S., Wang J., Wang S. (2020). Easy Green Construction of a Universal Sensing Platform Based on Crystalline Polyimide Covalent Organic Frameworks with Sensitive Fluorescence Response to Metal Ions and Antibiotics. ACS Appl. Bio Mater..

[2] Jia P., Bu T., Sun X., Liu Y., Liu J., Wang Q., Shui Y., Guo S., Wang L. (2019). A sensitive and selective approach for detection of tetracyclines using fluorescent molybdenum disulfide nanoplates. Food Chem..

[3] Yuan C., He Z., Chen Q., Wang X., Zhai C., Zhu M. (2021). Selective and efficacious photoelectrochemical detection of ciprofloxacin based on the self-assembly of 2D/2D g-C3N4/Ti3C2 composites. Appl. Surf. Sci..

[4] Yuan Y., Zhang F., Wang H., Gao L., Wang Z. (2018). A Sensor Based on Au Nanoparticles/Carbon Nitride/Graphene Composites for the Detection of Chloramphenicol and Ciprofloxacin. ECS J. Solid State Sci. Technol..

[5] Wang B., Pang M., Zhao X., Xie K., Zhang P., Zhang G., Zhang T., Liu X., Dai G. (2019). Development and comparison of liquid–liquid extraction and accelerated solvent extraction methods for quantitative analysis of chloramphenicol, thiamphenicol, florfenicol, and florfenicol amine in poultry eggs. J. Mass Spectrom..

[6] Xu X., Yang Y., Jin H., Pang B., Yang R., Yan L., Jiang C., Shao D., Shi J. (2020). Fungal In Situ Assembly Gives Novel Properties to CdSxSe1–x Quantum Dots for Sensitive Label-Free Detection of Chloramphenicol. ACS Sustain. Chem. Eng..

[7] Xie Y., Zhao M., Hu Q., Cheng Y., Guo Y., Qian H., Yao W. (2017). Selective detection of chloramphenicol in milk based on a molecularly imprinted polymer–surface-enhanced Ramanspectroscopic nanosensor. J. Raman Spectrosc..

[8] Hussain A., Alajmi M.F., Ali I. (2016). Determination of chloramphenicol in biological matrices by solid-phase membrane micro-tip extraction and capillary electrophoresis. Biomed. Chromatogr..

[9] Liu S., Wu X.-Z., Gao Z.-H., Jiao F. (2013). On-site solid phase extraction and HPLC determination of chloramphenicol in surface water and sewage. Anal. Methods.

[10] Barveen N.R., Wang T.-J., Chang Y.-H. (2021). Photochemical decoration of silver nanoparticles on silver vanadate nanorods as an efficient SERS probe for ultrasensitive detection of chloramphenicol residue in real samples. Chemosphere.

[11] Fedeniuk R.W., Mizuno M., Neiser C., O’Byrne C. (2015). Development of LC–MS/MS methodology for the detection/determination and confirmation of chloramphenicol, chloramphenicol 3-O-β-d-glucuronide, florfenicol, florfenicol amine and thiamphenicol residues in bovine, equine and porcine liver. J. Chromatogr. B.

[12] Vivekanandan K., Swamy M.G., Prasad S., Mukherjee R. (2005). A simple method of isolation of chloramphenicol in honey and its estimation by liquid chromatography coupled to electrospray ionization tandem mass spectrometry. Rapid Commun. Mass Spectrom..

[13] Yang H.-W., Xu P., Ding B., Liu Z.-Y., Zhao X.-J., Yang E.-C. (2019). A Highly Stable Luminescent Eu-MOF Exhibiting Efficient Response to Nitrofuran Antibiotics through the Inner Filter Effect and Photoinduced Electron Transfer. Eur. J. Inorg. Chem..

[14] Zhao Y., Li D. (2020). Lanthanide-functionalized metal–organic frameworks as ratiometric luminescent sensors. J. Mater. Chem. C.

[15] Gan Z., Hu X., Xu X., Zhang W., Zou X., Shi J., Zheng K., Arslan M. (2021). A portable test strip based on fluorescent europium-based metal–organic framework for rapid and visual detection of tetracycline in food samples. Food Chem..

[16] Xing K., Fan R., Du X., Zheng X., Zhou X., Gai S., Wang P., Yang Y. (2019). Dye-insertion dynamic breathing MOF as dual-emission platform for antibiotics detection and logic molecular operation. Sens. Actuators B Chem..

[17] Zhang L., Wang Y., Jia L., Bi N., Bie H., Chen X., Zhang C., Xu J. (2021). Ultrasensitive and visual detection of tetracycline based on dual-recognition units constructed multicolor fluorescent nano-probe. J. Hazard. Mater..

[18] Ti M., Li Y., Li Z., Zhao D., Wu L., Yuan L., He Y. (2021). A ratiometric nanoprobe based on carboxylated graphitic carbon nitride nanosheets and Eu3+ for the detection of tetracyclines. Analyst.

[19] Hu J., Yang X., Peng Q., Wang F., Zhu Y., Hu X., Zheng B., Du J., Xiao D. (2020). A highly sensitive visual sensor for tetracycline in food samples by a double-signal response fluorescent nanohybrid. Food Control.

[20] Ye Y., Wu T., Jiang X., Cao J., Ling X., Mei Q., Chen H., Han D., Xu J.J., Shen Y. (2020). Portable Smartphone-Based QDs for the Visual Onsite Monitoring of Fluoroquinolone Antibiotics in Actual Food and Environmental Samples. ACS Appl. Mater. Interfaces.

[21] Yan B. (2021). Luminescence response mode and chemical sensing mechanism for lanthanide-functionalized metal–organic framework hybrids. Inorg. Chem. Front..

[22] Zhao Y., Zeng H., Zhu X.-W., Lu W., Li D. (2021). Metal–organic frameworks as photoluminescent biosensing platforms: Mechanisms and applications. Chem. Soc. Rev..

[23] Zhong W.-B., Li R.-X., Lv J., He T., Xu M.-M., Wang B., Xie L.-H., Li J.-R. (2020). Two isomeric In(iii)-MOFs: Unexpected stability difference and selective fluorescence detection of fluoroquinolone antibiotics in water. Inorg. Chem. Front..

[24] Li C., Zhang F., Li X., Zhang G., Yang Y. (2019). A luminescent Ln-MOF thin film for highly selective detection of nitroimidazoles in aqueous solutions based on inner filter effect. J. Lumin..

[25] Mao X., Liu S., Su B., Wang D., Huang Z., Li J., Zhang Y. (2020). Luminescent europium(III)-organic framework for visual and on-site detection of hydrogen peroxide via a tablet computer. Microchim. Acta.

[26] Yang Y., Zhao L., Sun M., Wei P., Li G., Li Y. (2020). Highly sensitive luminescent detection toward polytypic antibiotics by a water-stable and white-light-emitting MOF-76 derivative. Dye. Pigment..

[27] Guo X., Pan Q., Song X., Guo Q., Zhou S., Qiu J., Dong G. (2020). Embedding carbon dots in Eu 3+ -doped metal-organic framework for label-free ratiometric fluorescence detection of Fe 3+ ions. J. Am. Ceram. Soc..

[28] Xiao J., Liu M., Tian F., Liu Z. (2021). Stable Europium-based Metal–Organic Frameworks for Naked-eye Ultrasensitive Detecting Fluoroquinolones Antibiotics. Inorg. Chem..

[29] Yu M., Xie Y., Wang X., Li Y., Li G. (2019). Highly Water-Stable Dye@Ln-MOFs for Sensitive and Selective Detection toward Antibiotics in Water. ACS Appl. Mater. Interfaces.

[30] Zheng L., Zhu T., Xu W., Liu L., Zheng J., Gong X., Wudl F. (2018). Solution-processed broadband polymer photodetectors with a spectral response of up to 2.5 μm by a low bandgap donor–acceptor conjugated copolymer. J. Mater. Chem. C.

[31] Wang B., Lv X.-L., Feng D., Xie L.-H., Zhang J., Li M., Xie Y., Li J.-R., Zhou H.-C. (2016). Highly Stable Zr(IV)-Based Metal–Organic Frameworks for the Detection and Removal of Antibiotics and Organic Explosives in Water. J. Am. Chem. Soc..

[32] Yang M., Tang Q., Meng Y., Liu J., Feng T., Zhao X., Zhu S., Yu W., Yang B. (2018). Reversible “Off–On” Fluorescence of Zn2+-Passivated Carbon Dots: Mechanism and Potential for the Detection of EDTA and Zn2+. Langmuir ACS J. Surf. Colloids.

[33] Miao X., Yan X., Qu D., Li D., Tao F.F., Sun Z. (2017). Red Emissive Sulfur, Nitrogen Codoped Carbon Dots and Their Application in Ion Detection and Theraonostics. ACS Appl. Mater. Interfaces.

[34] Lim S.A., Ahmed M.U. (2016). A Simple DNA-based Electrochemical Biosensor for Highly Sensitive Detection of Ciprofloxacin Using Disposable Graphene. Anal. Sci. Int. J. Jpn. Soc. Anal. Chem..

[35] Xia H., Peng M., Li N., Liu L. (2020). CdSe quantum dots-sensitized FRET system for ciprofloxacin detection. Chem. Phys. Lett..

[36] Garrido J.M.P.J., Melle-Franco M., Strutyński K., Borges F., Brett C.M.A., Garrido E.M.P.J. (2017). β–Cyclodextrin carbon nanotube-enhanced sensor for ciprofloxacin detection. J. Environ. Sci. Health Part A.

[37] Attia M.S., Youssef A.O., Ismael A.M., Gaafer R., Adel A., Twfik A., Wafeey A., Afify H.G., Sayed A. (2018). Highly Sensitive Eu^3+^ Doped in Sol-Gel Matrix Optical Sensor for The Assessment of Ciprofloxacin in Different Real Samples. Egypt J. Chem..

[38] Chang C.-C., Wang G., Takarada T., Maeda M. (2017). Iodine-Mediated Etching of Triangular Gold Nanoplates for Colorimetric Sensing of Copper Ion and Aptasensing of Chloramphenicol. ACS Appl. Mater. Interfaces.

[39] Li Y., Dai H., Feng N., Xie X., Zhang J., Li W. (2019). Silver chloride nanoparticles-decorated molybdenum disulfide nanosheets for highly sensitive chloramphenicol detection. Mater. Express.

