# Supplementary material for: Fluorescent Sensing of Ciprofloxacin and Chloramphenicol in Milk Samples via Inner Filter Effect and Photoinduced Electron Transfer Based on Nanosized Rod-Shaped Eu-MOF
Source: Foods. 2022 Oct 9;11(19):3138. doi: 10.3390/foods11193138 (PMC9562874; doi:10.3390/foods11193138)
Supplement: Supplementary file 1 [file foods-11-03138-s001.zip › foods-1944355-supplementary.pdf]

Fluorescent Sensing of Ciprofloxacin and Chloramphenicol in milk samples via Inner Filter Effect (IFE) and Photoinduced Electron Transfer based on nanosized rod-shaped Eu-MOF

Xiaoyue Yue [1,3], Chaoyun Wu[1], Zijun Zhou [2], Long Fu [1], Yanhong Bai [1,3,*]

[1] College of Food and Bioengineering, Zhengzhou University of Light Industry, Zhengzhou 450001, China

[2] Henan Institute of Product Quality Supervision and Inspection, Zhengzhou 450001, China

[3] Henan Key Laboratory of Cold Chain Food Quality and Safety Control, Zhengzhou 450001, China

* Correspondence: baiyanhong212@163.com

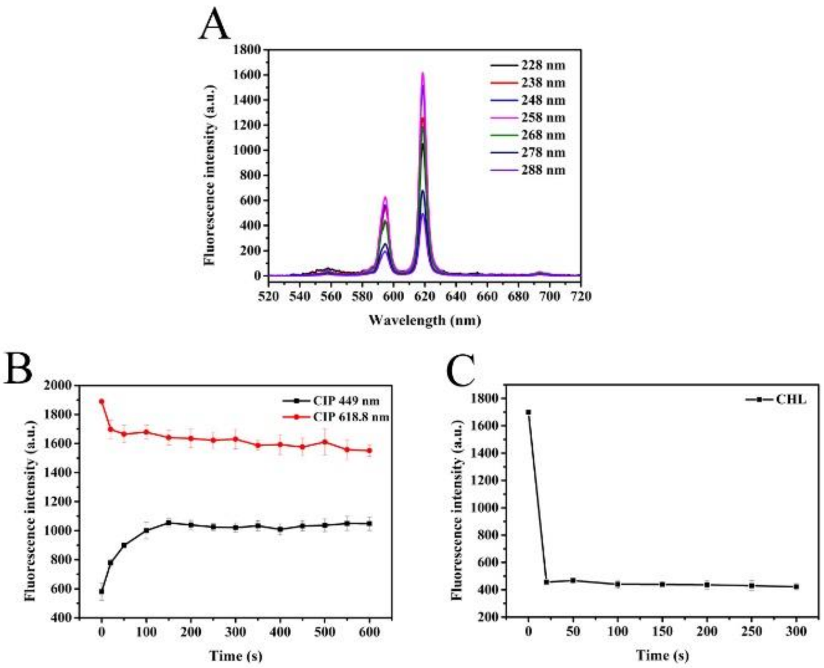

Figure. S1 (A) Fluorescence emission spectra of Eu-MOF at different excitation wavelengths; (B) Fluorescence intensity of Eu-MOF at 449 nm and 618.8 nm after the addition of ciprofloxacin with time; (C) Fluorescence intensity of Eu-MOF at 618.8 nm after the addition of chloramphenicol with time

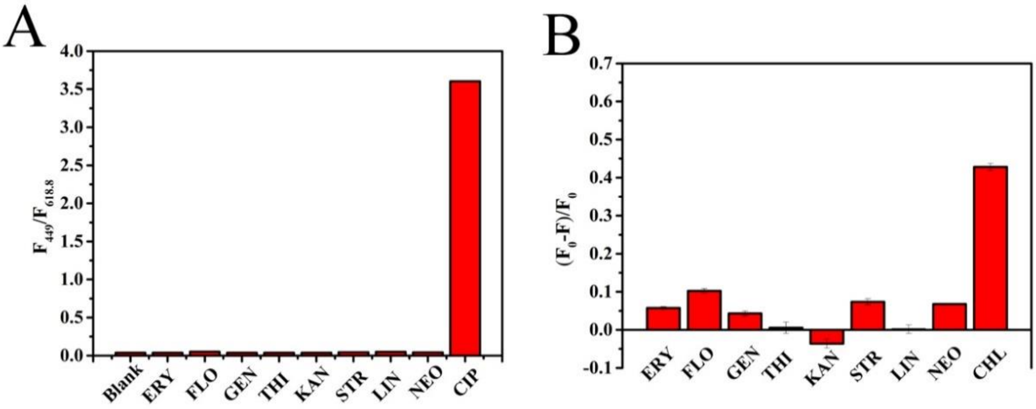

Figure. S2. (A) The $F_{449}/F_{618.8}$ and (B) $(F_0\text{-}F)/F_0$ at 618.8nm of the Eu-MOF against various antibiotics.
